# Characterization and Pathogenicity of *Colletotrichum truncatum* Causing *Hylocereus undatus* Anthracnose through the Changes of Cell Wall-Degrading Enzymes and Components in Fruits

**DOI:** 10.3390/jof10090652

**Published:** 2024-09-13

**Authors:** Shuwu Zhang, Yun Liu, Jia Liu, Enchen Li, Bingliang Xu

**Affiliations:** 1 Biocontrol Engineering Laboratory of Crop Diseases and Pests of Gansu Province, College of Plant Protection, Gansu Agricultural University, Lanzhou 730070, China; zhangsw704@126.com (S.Z.); l2046584054@163.com (Y.L.); jiajia7635724@163.com (J.L.); liec188@163.com (E.L.); 2 Gansu Provincial Key Laboratory of Arid Land Crop Science, Gansu Agricultural University, Lanzhou 730070, China

**Keywords:** dragon fruit, anthracnose, pathogens identification, cell wall-degrading enzymes, cell wall components

## Abstract

Anthracnose is one of the destructive diseases of pitaya that seriously affects the plant growth and fruit quality and causes significant yield and economic losses worldwide. However, information regarding the species of pathogens that cause anthracnose in pitaya (*Hylocereus undatus*) fruits in Gansu Province, China, and its pathogenic mechanism is unknown. Thus, the purposes of our present study were to identify the species of pathogens causing *H. undatus* fruits anthracnose based on the morphological and molecular characteristics and determine its pathogenic mechanism by physiological and biochemical methods. In our present study, forty-six isolates were isolated from the collected samples of diseased *H. undatus* fruits and classified as three types (named as H-1, H-2, and H-3), according to the colony and conidium morphological characteristics. The isolation frequencies of H-1, H-2, and H-3 types were 63.04%, 21.74%, and 15.22%, respectively. The representative single-spore isolate of HLGTJ-1 in H-1 type has significant pathogenicity, and finally we identified *Colletotrichum truncatum* as the pathogen based on the morphological characteristics as well as multi-locus sequence analysis. Moreover, the *H. undatus* fruits inoculated with *C. truncatum* had a significantly increased activity of cell wall-degrading enzymes (CWDEs) cellulase (Cx), β-glucosidase (β-Glu), polygalacturonase (PG), and pectin methylgalacturonase (PMG), while having a decreased level of cell wall components of original pectin and cellulose in comparison to control. The average increased activities of Cx, β-Glu, PG, and PMG were 30.73%, 40.40%, 51.55%, and 32.23% from day 0 to 6 after inoculation, respectively. In contrast, the average decreased contents of original pectin and cellulose were 1.82% and 16.47%, respectively, whereas the average increased soluble pectin content was 38.31% in comparison to control. Our results indicate that *C. truncatum* infection increased the activities of CWDEs in *H. undatus* fruits to disassemble their cell wall components, finally leading to the fruits’ decay and deterioration. Thus, our findings will provide significant evidence in the controlling of pitaya anthracnose in the future.

## 1. Introduction

Pitaya (*Hylocereus* spp.) is known as a representative and important tropical and sub-tropical fruit due to its high nutrients, medicinal properties, and high economic values [1,2,3]. Now, it is widely cultivated in America, Vietnam, China, Mexico, Nicaragua, Malaysia, Colombia, Thailand, Ecuador, and other countries [4]. However, with increasing of cultivation area, pitaya fruit diseases are becoming a key problem, resulting in the fruits’ decay, shortened shelf life, and economic losses [5]. Anthracnose caused by *Colletotrichum* spp. are one of the most devastating preharvest and postharvest diseases of pitaya fruits [6,7,8]. Some studies reported that the disease incidence of pitaya anthracnose was 50% in an orchard in Fortaleza, Ceará, Brazil, in 2018 [9], and also up to 35% in the municipality of Pelotas, Rio Grande do Sul state, Brazil, in 2017 [10]. The yield losses due to it in pitaya were up to 20–80% in Malaysia [11] and approximately 50% losses in pitaya were caused by *Colletotrichum gloeosporioides* [12]. In 2020, we found that anthracnose occurred in the greenhouse of pitaya (*H. undatus*) fruits in Wuwei city, Gansu Province, China, with a field incidence of 15.6%. However, the species of pathogens that cause pitaya anthracnose are different in different regions, and related work on species identification is still lacking in the world. In previous studies, the species of pathogens causing pitaya fruit anthracnose have been reported as *C. gloeosporioides* (Malaysia, USA, Japan, and Brazil) [11,13,14,15], *C. truncatum* (Malaysia and India) [16,17] and *C. siamense* (India) [18], *C. tropicale* (Mexico) [19], *C. karstii* (Brazil) [10], *C. aenigma* and *C. siamense* (Thailand) [20], and *C. fructicola* (Philippines) [21]. In China, the species of *C. gloeosporioides* [22], *C. siamense* [23], and *C. truncatum* [24] that cause pitaya fruit anthracnose have been reported in Guangzhou Province, Hainan Province, Yunnan Province, and other regions. Meanwhile, the species of *C. gloeosporioides*, *C. truncatum*, and *C. boninense* that can cause *H. polyrhizus*, *H. undatus*, and *H. costaricensis* anthracnose have been reported in Taiwan [25]. However, information on the pathogens of pitaya fruit anthracnose in China is still lacking, especially information regarding the species of pathogens that cause anthracnose in *H. undatus* fruits in Gansu Province, China, and their pathogenic mechanisms remains unknown.

As is well known, the cell wall is the first mechanical barrier for the plants against fungal pathogen invasion and infection [26], but the cell wall-degrading enzymes (CWDEs) that are secreted by different species of *Colletotrichum* play a key role in penetrating the barrier of the plant cell wall [27], infecting the plant tissue, and leading to the infected tissue’s death, maceration, decay, and deterioration [28,29]. Similarly, we have found that the pathogenic isolate can cause the *H. undatus* fruit’s tissue maceration and decay, even resulting in the whole fruits softening finally in greenhouse or pathogenicity testing in the present study. Moreover, a previous study reported that the gradually increased activities of CWDEs (polygalacturonase (PG), pectin polysaccharide (PME), β-galactosidase, and cellulase) play the important role in modifying the pectic and hemi-cellulosic components in the cell wall of pitaya fruits during fruit softening [30]. To date, there is still a lack of research regarding the effect of *Colletotrichum* sp. infection on the activities of the CWDEs and the contents of pectin and cellulase in pitaya fruits.

Thus, the aims of our present study were to identify the species of the pathogens causing *H. undatus* fruit anthracnose in Gansu Province, China, based on the morphological and molecular characteristics, and investigate the impact of the pathogens on the changes in CWDE activities and cell wall components in *H. undatus* fruits.

## 2. Materials and Methods

### 2.1. Fruit Sample Collection

In 2020, twenty-eight *Hylocereus undatus* fruit samples with typical anthracnose symptoms were collected from the greenhouse in Wuwei city, Gansu Province, China. The diseased fruit samples were labeled and stored at 4 °C for pathogen isolation.

### 2.2. Pathogen Isolation and Purification

The pathogens were isolated from *H. undatus* fruits with typical anthracnose symptoms by the method of tissue isolation. The diseased fruit samples were rinsed with sterile water three times and then dried using sterile paper for isolating the pathogens. The fruit sections were prepared by cutting the junction between the diseased and healthy fruit areas (0.5 cm long × 0.5 cm wide), and then disinfecting with 75% ethanol for 30 s, followed by rinsing with sterile water three times. Thereafter, the sterilized fruits sections were dried on the sterile paper and then inoculated with potato dextrose agar (PDA) medium with five sections for each. The inoculation PDA plates were incubated with a constant temperature of 25 °C and light/dark (12 h/12 h) for 3 days. Isolates with different morphological characteristics of colony and conidium were purified by cutting the mycelial discs (d = 0.5 cm) from the edge of colony at 3 days after incubation, and inoculated on the center of new PDA medium for 7 days. Finally, the single-spore isolates were purified by inoculating the single spore from the 7-day-old isolate colony on the PDA media. The morphological characteristics of the single-spore isolates were recorded, and their isolation frequencies were calculated at 7 days after incubation. The representative single-spore isolates were also used for further pathogenicity determination and morphological and molecular characteristics identification.

### 2.3. Isolate Pathogenicity Determination

For the pathogenicity testing, three representative isolates were selected from types H-1, H-2, and H-3 (one isolate for each type), named as HLGTJ-1, HLGTJ-2, and HLGTJ-3. The pathogenicity of the representative single-spore isolates was determined by inoculating the mycelial discs of the isolates on the detached and healthy *H. undatus* fruit samples according to Koch’s postulates. The uniform maturity and healthy *H. undatus* fruits were disinfected with 75% ethanol for 30 s, and then rinsed with sterile water three times and air-dried on a bench for pathogenicity testing. The specific methods for the representative single-spore isolate’s pathogenicity testing are described by Zhang et al. (2022) [31]. The fruits inoculated with the mycelial discs of the isolates and the PDA medium discs without mycelia were regarded as the treatment and control, respectively. Thereafter, the inoculation fruit samples were placed in a plastic container at a constant temperature of 25 °C with 60% relative humidity, and incubated under a light/dark (12 h/12 h) photoperiod. The pathogenicity and symptoms of the inoculation fruits were determined and observed from 1 to 7 days after the inoculation for 2 days internally. At day 7, the pathogenic isolates were re-isolated and incubated on PDA media to observe the morphological characteristics in comparison to the inoculation isolates. The experiments in the treatment and control group were repeated three times, and three fruit samples were used for each replicate and representative isolate, respectively.

### 2.4. Morphological Characteristics of the Isolate Identification

The diameter of mycelial discs (d = 0.5 cm) were cut from the edge of the 7-day-old single-spore isolate colony, inoculated individually on the center of PDA media, and incubated at 25 °C. The morphological characteristics of the colony, acervulus, conidium, seta, appressorium, and number of acervuli and setae formed were observed and recorded. Additionally, the sizes of acervulus, seta, and conidium samples (100 for each replicate), and appressorium (50 for each replicate) were measured for each of the three replicates, and then the mean value was determined and calculated for the isolate. Finally, the morphological characteristics of the isolate was identified and based primarily on the species description by Damm et al. (2009) [32] and Guo et al. (2014) [24] in previous studies.

### 2.5. Molecular Characteristics of the Isolate Identification

For the identification of the isolate species, the fresh mycelia of the representative single-spore isolate were collected at 7 days after incubation on PDA media, and quickly frozen with liquid nitrogen for the genomic DNA extraction. The genomic DNA was extracted by the modified CTAB method and stored in the refrigerator at −20 °C for further experiment. The genomic DNA of the representative single-spore isolate was used as a template to amplify the target internal transcribed spacers (ITSs), glyceraldehyde-3-phosphate dehydrogenase (*GAPDH*), and histone 3 (*HIS3*) gene fragments by using the Polymerase Chain Reaction (PCR) amplification. The sequences of the ITS region and the *GAPDH* and *HIS3* genes of the representative single-spore isolate were amplified using the primers of HJ-ITS5/HJ-ITS4, *HJ-GAPDHF*/*HJ-GAPDHR,* and *HJ-HIS3F*/*HJ-HIS3R* [33,34] (Table 1). The primers and PCR products used in the present study were synthesized and sequenced by Sangon Biotech Co., Ltd. (Shanghai, China), respectively. The sequences of the HJ-ITS region and *HJ-GAPDH* and *HJ-HIS3* genes were aligned on the NCBI website using BLAST search, and then the related sequences were downloaded and submitted to multi-locus phylogenetic analyses based on maximum likelihood (ML) by combining the dataset of HJ-ITS region and *HJ-GAPDH* and *HJ-HIS3* sequences. Multi-locus phylogenetic tree construction was performed using the IQ-TREE (v1.6.12) software, with a boot-strap value of 1000.

### 2.6. Effect of Isolate Infection on the Physiological-Biochemical Characteristics of H. undatus Fruits

The uniformly mature and healthy *H. undatus* fruits with the same size were disinfected with 75% ethanol for 2 min and finally rinsed with sterile water and air-dried by placing on a bench for inoculating. The mycelial discs (d = 0.5 cm) were prepared at the edge of the isolate colony at 7 days after inoculation. The specific method for the inoculation is the same as the pathogenicity of isolate determination. Each fruit was inoculated with three mycelial discs (treatment) or the PDA medium discs without mycelia (control). After inoculation, the fruits in the treatment and control group were placed in a plastic container and incubated at 25 °C in an incubator with a constant relative humidity of 60% and a light/dark (12 h/12 h) photoperiod. For the determination of the effect of the isolate infection on the physiological-biochemical characteristics of *H. undatus* fruits, the junction tissues between the diseased and healthy fruit area in the treatment fruits and the junction tissues between the inoculation site and healthy fruit area in the control fruits were collected at 0, 1, 2, 3, 4, 5, and 6 days after inoculation. Each experiment in the treatment and control group was repeated three times and with three *H. undatus* fruits in each replicate.

#### 2.6.1. Effect of the Isolate Infection on the Activities of Cell Wall-Degrading Enzymes of *H. undatus* Fruits

The methods for the extraction and activity determination of the CWDEs cellulase (Cx), β-glucosidase (β-Glu), polygalacturonase (PG), and pectin methylgalacturonase (PMG) were according to the instructions for the plant CWDEs assay kit (Shanghai Optimal Biotechnology Co., Ltd., Shanghai, China), and three replicates were set for each treatment and control.

#### 2.6.2. Effect of the Isolate Infection on the Contents of Cell Wall Components of *H. undatus* Fruits

The cell wall components of original pectin, soluble pectin, and cellulose content in *H. undatus* fruits were determined according to the instructions for the plant cell wall components assay kit (Shanghai Optimal Biotechnology Co., Ltd., Shanghai, China), and three replicates were set for each treatment and control.

### 2.7. Statistical Analysis

The experiments in the present study were repeated three times, and data were the mean of the three replicates. The values of CWDEs activities and cell wall components in *H*. *undatus* fruits at different days after inoculation with the isolate or not were expressed as mean and standard errors. One-way ANOVA analysis was carried out and tested for the significant differences among the treatments using statistical analysis system SPSS version 16.0 (SPSS Inc., Chicago, IL, USA), and Duncan’s multiple range test at *p* < 0.05.

## 3. Results

### 3.1. Observation of the Symptoms of H. undatus Fruits Anthracnose in Greenhouse

**Figure 1:**
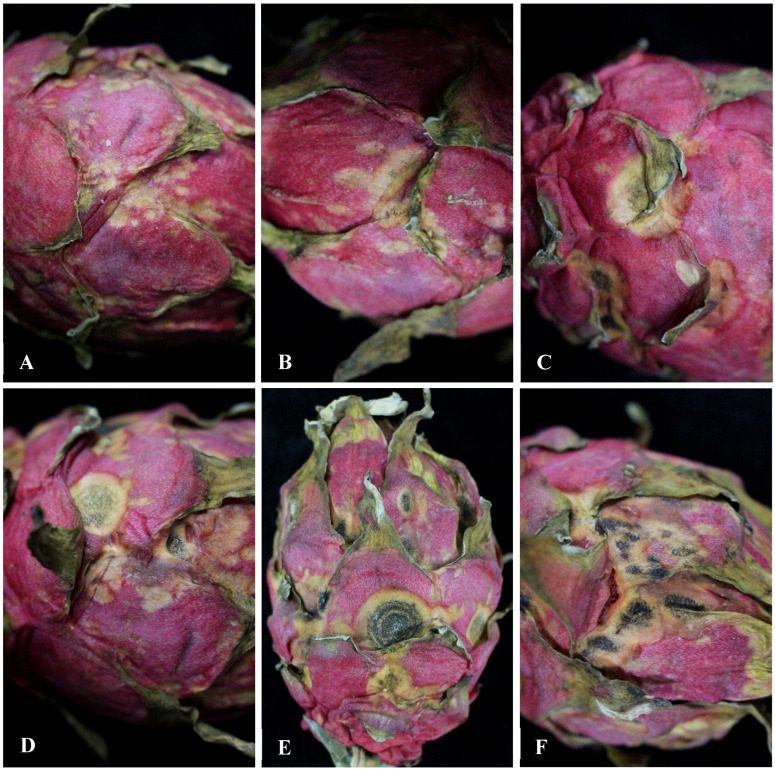
Symptoms of *Hylocereus undatus* fruit anthracnose at different time periods in Wuwei city, China. (**A**) and (**B**): the symptoms at the initial stage; (**C**–**F**): the symptoms at a later stage.

The symptoms initially observed in the greenhouse appeared as reddish-orange spots, which later became larger and changed to pale brown lesions in *H. undatus* fruits (Figure 1A,B). As the lesions progressed, the centers of the lesions were gray-white with brown border (Figure 1C), and a large number of black dots (black acervuli) were formed (some with concentric circles) on the lesions center of *H. undatus* fruits (Figure 1D–F).

### 3.2. Isolation and Determination of the Pathogenicity of the Isolates

Forty-six isolates were isolated from the collected samples of the diseased *H. undatus* fruits, and primary classified as three types according to the colony and conidium morphological characteristics (named as H-1, H-2, and H-3). Among the 46 isolates, 29, 10, and 7 isolates were identified as types H-1, H-2, and H-3, respectively. The isolation frequencies of the H-1, H-2, and H-3 types were 63.04%, 21.74%, and 15.22%, respectively.

**Figure 2:**
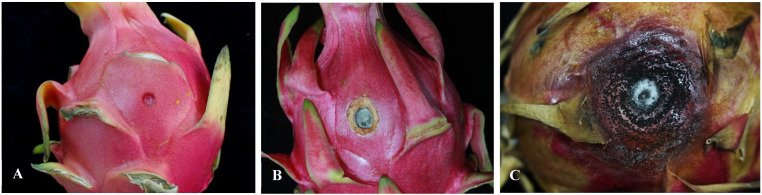
The pathogenicity test of the representative isolate of HLGTJ-1 on *Hylocereus undatus* fruit after inoculation. (**A**) Fruit inoculation with the PDA discs without the HLGTJ-1 isolate (control); (**B**,**C**) fruits inoculation with the mycelial discs of the HLGTJ-1 isolate at 3 and 7 days after inoculation, respectively.

The in vitro pathogenicity testing of the representative isolate HLGTJ-1 resulted in significant symptoms on the inoculated fruits that were similar to the anthracnose symptoms found in the greenhouse among the representative isolates HLGTJ-1, HLGTJ-2, and HLGTJ-3. The brown lesion formed on the inoculated area of the fruit with white or light grey mycelia on the center at 3 days after inoculation (Figure 2B). Subsequently, with the continuous expansion of the lesion, typical anthracnose symptoms of the disease lesion occurred at a later stage after inoculation. The lesion center became gray-white, and a large number of black dots formed on the surface of the lesion at 7 days (Figure 2C). In contrast, the fruit in the control group did not show any disease symptoms (Figure 2A). The morphology of the re-isolates from the infected fruit was consistent with the inoculated isolate, while no isolates were isolated from the control fruit. Thus, the representative isolate of HLGTJ-1 in the H1 type was identified as the pathogenic isolate that causes *H. undatus* fruit anthracnose according to Koch’s postulates.

### 3.3. Morphological Characteristics of the Isolate

**Figure 3:**
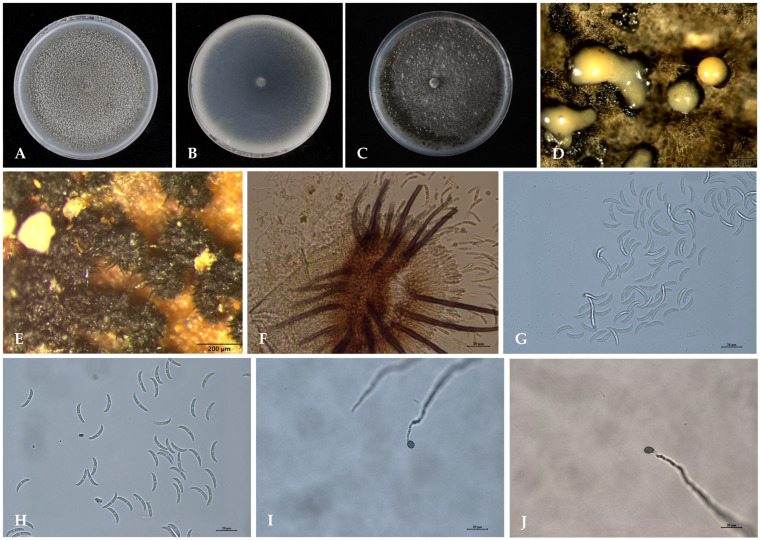
Morphological characteristics of the representative isolate of HLGTJ-1. (**A**,**B**) The front and reverse views of the colony, respectively; (**C**) the conidial mass produced on the front of the colony; (**D**) the conidial mass observed under a stereoscope; (**E**) the acervuli and setae observed under a stereoscope; (**F**) the acervulus and setae observed under a microscope using the hand-sliced method; (**G**,**H**) conidia; (**I**,**J**) appressorium.

The colony of HLGTJ-1 on PDA medium exhibited white to light grey mycelia initially, and then changed to dark grey in the front, whereas thick dark grey mycelia with distinct zonation was observed in the reverse of the plate. Thereafter, the colony on PDA medium exhibited abundant black acervuli (Figure 3A) and pinkish-orange conidial mass (Figure 3C,D) in the front, while dark gray color was observed in the reverse of the plate (Figure 3B) after incubation. The acervulus was round or oval, and dark brown (238.05 × 457.49 μm) (Figure 3E,F). The seta was dark brown to black, rigid and straight, with a swollen base and a tapered apex (Figure 3E,F). The conidium (21.27 μm × 4.04 μm) was colorless, crescent or sickle-shaped, with an acute apex and a narrow truncate base (Figure 3G,H). A single germ tube was germinated from the middle or top of each conidium and formed a spherical to ovate appressorium (8.13 μm × 11.75 μm) (Figure 3I,J). Finally, the morphological characteristics indicates that the HLGTJ-1 isolate had the same characteristics as previously described for *Colletotrichum truncatum*.

### 3.4. Molecular Identification of the Isolate

**Figure 4:**
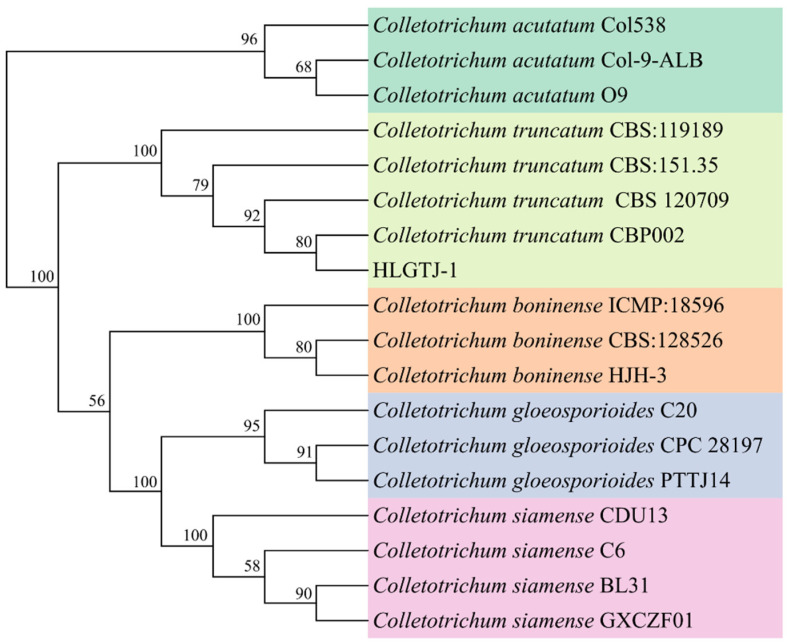
Multi-locus phylogenetic tree of the single-spore isolate of HLGTJ-1 based on the combined sequences (HJ-ITS region and *HJ-GAPDH* and *HJ-HIS3* genes) by the maximum likelihood (ML) method. Bootstraps supporting values higher than 50% from the 1000 replicates are presented at the nodes.

The fragments of the HJ-ITS region and *HJ-GAPDH* and *HJ-HIS3* genes were obtained by PCR amplification, and the sequences of the HJ-ITS region and *HJ-GAPDH* and *HJ-HIS3* genes were more than 99% identical to *Colletotrichum truncatum* previously deposited in the NCBI database. The combined dataset of the HJ-ITS region and *HJ-GAPDH* and *HJ-HIS3* genes sequences of the HLGTJ-1 isolate was grouped within the *C. truncatum* CBP002 (KF300886.1) clade and with support rates of 80% (Figure 4). Thus, the isolate of HLGTJ-1 was identified as *C. truncatum*, based on the multi-locus sequences of the HJ-ITS region and *HJ-GAPDH* and *HJ-HIS3* genes.

### 3.5. Effect of Isolate Infection on the Activities of Cell Wall-Degrading Enzymes of H. undatus Fruits

**Figure 5:**
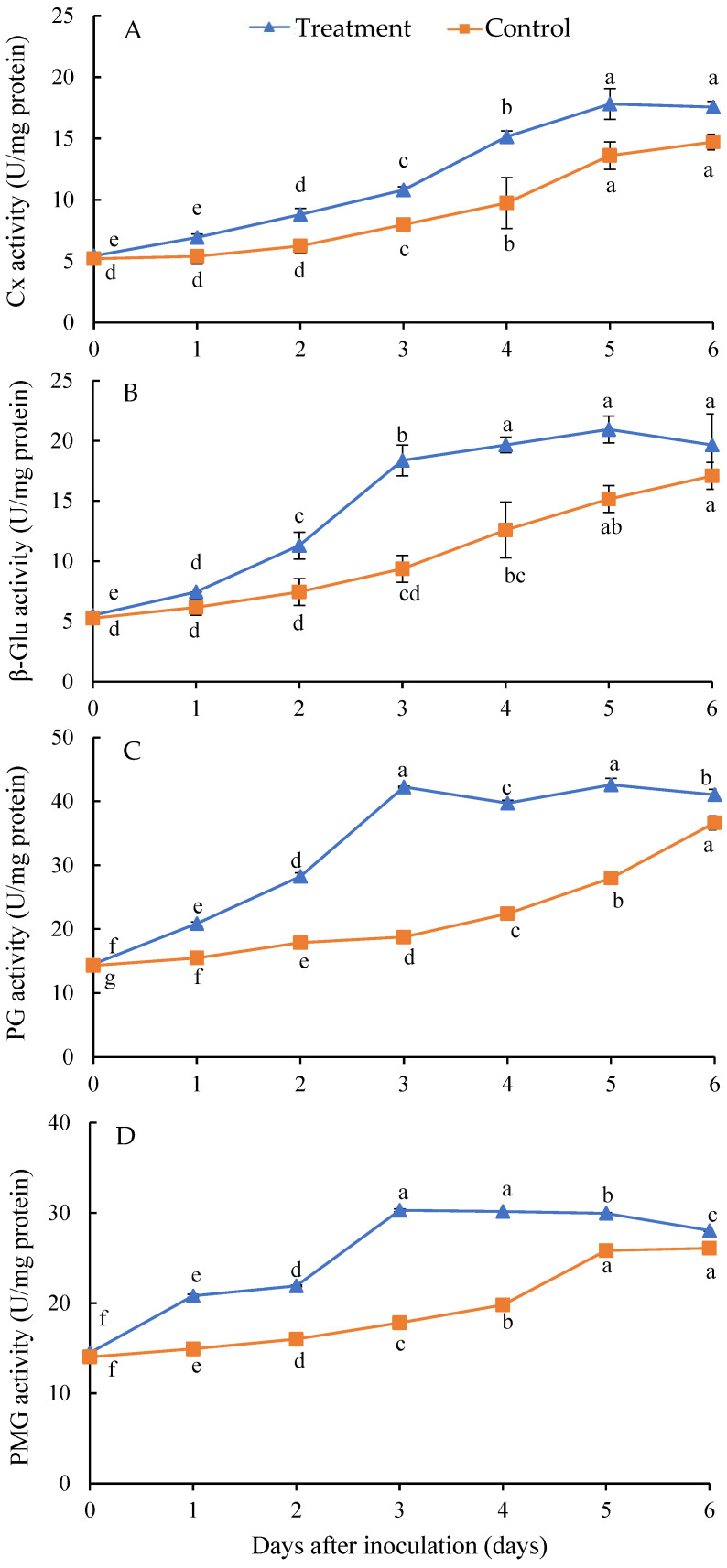
Changes in activities of Cx (**A**), β-Glu (**B**), PG (**C**), and PMG (**D**) in *Hylocereus undatus* fruits at different days after inoculation with the isolate. Different letters in Figure are significantly different at *p* < 0.05.

The activities of Cx (Figure 5A) and β-Glu (Figure 5B) in the healthy and diseased fruits’ tissue showed a rapid increase from day 0 to 5 after inoculation, whereas the activity of PG (Figure 5C) and PMG (Figure 5D) showed a rapid increase from day 0 to 3 after inoculation. The average activities of Cx (Figure 5A), β-Glu (Figure 5B), PG (Figure 5C), and PMG (Figure 5D) were significantly increased from day 0 to 6 after inoculation with the isolate in comparison to the control healthy fruits. Compared with the control, the average increased activities of Cx, β-Glu, PG, and PMG were 30.73%, 40.40%, 51.55%, and 32.23% from day 0 to 6 after inoculation, respectively. In addition, the activities of Cx and β-Glu reached a peak on the fifth day, whereas those of PG and PMG did so on the third day in the treatment group after inoculation.

### 3.6. Effect of Isolate Infection on the Contents of Cell Wall Components of H. undatus Fruits

**Figure 6:**
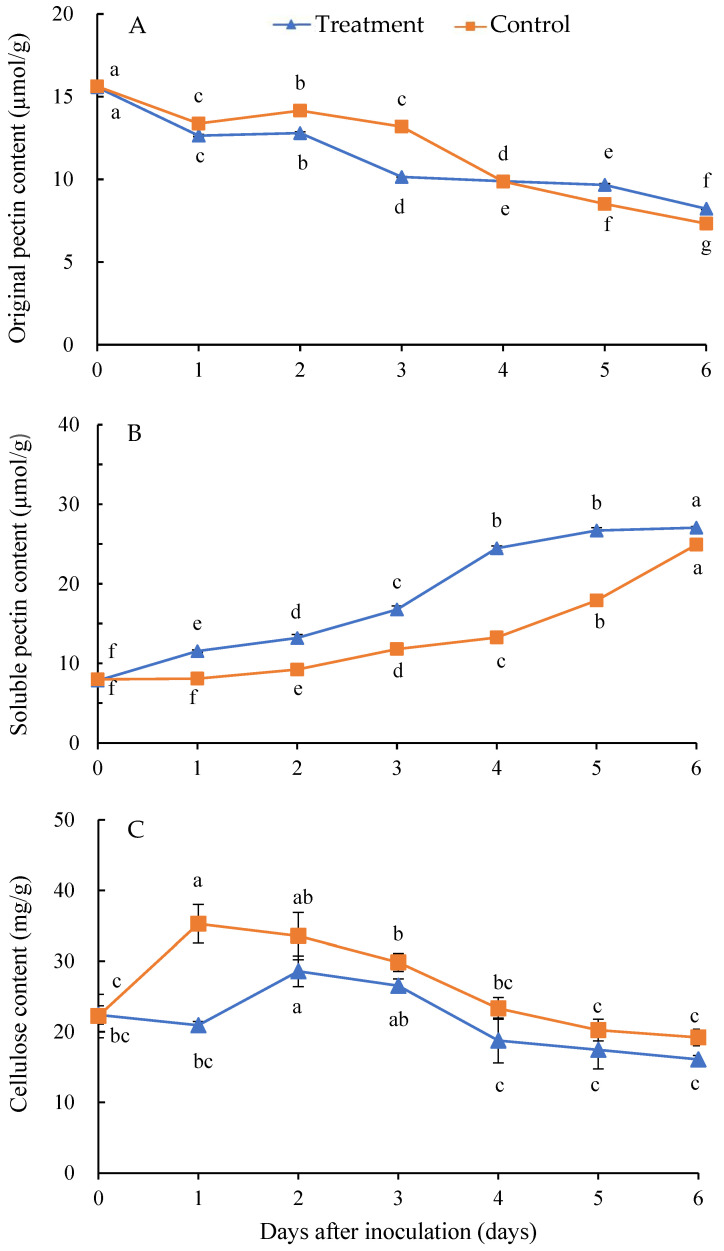
Changes in original pectin (**A**), soluble pectin (**B**), and cellulose (**C**) contents in *Hylocereus undatus* fruits at different days after inoculation with the isolate. Different letters in Figure are significantly different at *p* < 0.05.

Compared with the control, the original pectin content in the inoculated fruits’ tissue was decreased from day 0 to 3 after inoculation. Initially, the original pectin content gradually declined, followed by an increase and subsequent decrease. The average decrease in original pectin content was 1.82% from day 0 to 6 after inoculation in comparison to the control (Figure 6A). Conversely, the soluble pectin content was increased from day 0 to 6 in the control and treatment groups. Compared with the control, the average content of soluble pectin in the inoculated fruits’ tissue was significantly higher than the healthy fruits from day 0 to 6 after inoculation. The average increased content of soluble pectin was 38.31% from day 0 to 6 after inoculation in comparison to the control (Figure 6B).

The isolate infection had a significant effect on the cellulose content in *H. undatus* fruits. Compared with the control, the average cellulose content in the inoculated fruits’ tissue was significantly decreased from day 0 to 6 after inoculation. The peak of cellulose content was 35.28 and 28.58 mg/g in the control and treatment at 1 and 2 days after inoculation. The average decreased cellulose content was 16.47% from day 0 to 6 after inoculation in comparison to the control (Figure 6C).

## 4. Discussion

*Colletotrichum* spp. are some of the most prevalent fungal pathogens causing anthracnose in diverse tropical and subtropical fruits [35]. Seven species of *Colletotrichum* spp., including *C. gloeosporioides*, *C. aenigma*, *C. tropicale, C. siamense*, *C. truncatum*, *C. karstii,* and *C. fructicola*, that can cause pitaya anthracnose have been reported in Brazil, China, the US, Thailand, the Philippines, Malaysia, and other regions. Among the seven *Colletotrichum* species, *C. gloeosporioides* has been reported as the major anthracnose pathogen in *Hylocereus* spp. around the world [15], and several members within the *C. gloeosporioides* complex have been identified and reported by the application of multiple genes or regions. Recently, the species *C. truncatum* has been reported as causing anthracnose on different types of beans and lentils [36], guar [37], tomato [38], strawberry [39], papaya [40], and chilli [41], except the species within the *C. gloeosporioides* complex. In the present study, we discovered the species of *C. truncatum* causing *H. undatus* fruit anthracnose in Wuwei city, Gansu Province, China, according to the morphological characteristics and multi-locus sequence analysis of the ITS region and *GAPDH* and *HIS3* genes. Similarly, *C. truncatum* was reported as causing anthracnose in *H. polyrhizus* stem in Malaysia [16], *H. undatus* and *H. polyrhizus* plants in India [17], and *H. undatus* fruits in a market in Yunnan Province, China [24]. However, *C. truncatum* causing *H. undatus* fruit anthracnose at harvest time has never been reported in Gansu Province, China. To our knowledge, this is the first report of *C. truncatum* causing anthracnose on *H. undatus* fruits at harvest time in Gansu Province, China.

Furthermore, the activities of Cx, β-Glu, PG, and PMG in *H. undatus* fruits infected by *C. truncatum* were significantly higher than those in healthy fruits. Similarly, some studies revealed that the pathogen of *Phomopsis longanae* infection significantly increased the activities of the CWDEs pectinesterase, polygalacturonase, β-galactosidase in the pericarp of harvested “Fuyan” longan (*Dimocarpus longan* Lour. cv. *Fuyan*) fruits [42]; *Macrophomina phaseolina* infection can induce the cell wall degradation of maize and sunflower [43]. In addition, the substances pectin, hemicelluloses, and cellulose are the main components of the cell wall which contribute to the mechanical properties of the cell wall and the strength of the pericarp [44,45]. However, the degradation of pectin can disassemble the cellulose and hemicellulose in the cell wall, resulting in the tissue of pericarp loosening or the fruit softening [46,47,48]. Our results found that the soluble pectin content in the inoculated fruits significantly increased after inoculation, and was higher than that in the control fruits, whereas the original pectin and cellulose contents in the inoculated fruits were lower than those in the control fruits. Likewise, other studies revealed that the pathogenic *Botryodiplodia theobromae* caused mango stem-end rot through producing PG, PMG, and CX to disrupt the fruits’ tissue in the deterioration process [49]; *Phomopsis longanae* inoculation treatment accelerated the degradation of the cell wall components including chelate-soluble pectin, Na_2_CO_3_-soluble pectin, hemicellulose, and cellulose in the longan pericarp cell wall and middle lamella, whereas it elevated the water-soluble pectin content [42].

## 5. Conclusions

In the present study, *C. truncatum* was first reported as the agent that caused anthracnose in *H. undatus* fruits at harvest time in Gansu Province, China, based on the morphological characteristics as well as multi-locus sequence analysis. Moreover, the *H. undatus* fruits inoculated with *C. truncatum* had significantly increased activity of Cx, β-Glu, PG, and PMG, and content of soluble pectin, while the average contents of the cell wall components of cellulose and original pectin were decreased in comparison to the control. Our results indicate that *C. truncatum* infection can increase the activities of *H. undatus* fruit CWDEs to disassemble the cell wall components of original pectin and cellulose, finally leading to the infected fruits’ tissue death, maceration, decay, and deterioration.

## Figures and Tables

**Table 1:** Primers for the PCR amplification in the present study.

Region or Genes	Primer Name	Primer Sequence (5′–3′)	Annealing Temperature (°C)
ITS	HJ-ITS4 HJ-ITS5	TCCTCCGCTTATTGATATGC GGAAGTAAAAGTCGTAACAAGG	56
*GAPDH*	*HJ-GAPDHF* *HJ-GAPDHR*	GCCGTCAACGACCCCTTCATTG GGGTGGAGTCGTACTTGAGCAT	56
*HIS3*	*HJ-HIS3F* *HJ-HIS3R*	AGGTCCACTGGTGGCAAG AGCTGGATGTCCTTGGACTG	54

## Data Availability

The original contributions presented in the study are included in the article, and further inquiries can be directed to the corresponding author.
